# Meal Timing and Macronutrient Composition Modulate Human Metabolism and Reward-Related Drive to Eat

**DOI:** 10.3390/nu14030562

**Published:** 2022-01-27

**Authors:** Rodrigo Chamorro, Swantje Kannenberg, Britta Wilms, Christina Kleinerüschkamp, Svenja Meyhöfer, Soyoung Q. Park, Hendrik Lehnert, Henrik Oster, Sebastian M. Meyhöfer

**Affiliations:** 1 Institute for Endocrinology and Diabetes, University of Lübeck, Ratzeburger Allee 160, 23562 Lübeck, Germany; rchamorro@uchile.cl (R.C.); swantje.kannenberg@diabetes-luebeck.de (S.K.); britta.wilms@uni-luebeck.de (B.W.); chr.hoeber@t-online.de (C.K.); Svenja.Meyhoefer@uksh.de (S.M.); 2 Department of Nutrition, Faculty of Medicine, University of Chile, Santiago 8380453, Chile; 3 German Center for Diabetes Research, 85764 München-Neuherberg, Germany; soyoung.q.park@gmail.com; 4 Center of Brain, Behavior and Metabolism (CBBM), University of Lübeck, 23562 Lübeck, Germany; henrik.oster@uni-luebeck.de; 5 Department of Internal Medicine 1, Endocrinology & Diabetes, University Hospital Schleswig-Holstein Campus Lübeck, 23562 Lübeck, Germany; 6 Department of Decision Neuroscience and Nutrition, German Institute of Human Nutrition (DIfE), Potsdam-Rehbrücke, 14558 Nuthetal, Germany; 7 Charite-Universitatsmedizin Berlin, Corporate Member of Freie Universität Berlin, Humboldt-Universität zu Berlin, Berlin Institute of Health, Neuroscience Research Center, 10117 Berlin, Germany; 8 Paris Lodron University, 5020 Salzburg, Austria; hendrik.lehnert@sbg.ac.at; 9 Institute of Neurobiology, University of Lübeck, 23562 Lübeck, Germany

**Keywords:** meal timing, macronutrient composition, carbohydrates, hedonic eating, liking, wanting, circadian rhythm

## Abstract

The ‘time-of-day’ modifies the metabolic response to meals, but less data exist on the diurnal variations in the hedonic drive to eat. In the present paper, we evaluate the effects of meal timing and macronutrient composition on metabolic responses and the homeostatic vs. hedonic regulation of appetite. In study 1, 84 young, healthy adults completed an online computer-based task assessing the homeostatic and hedonic drive to eat in the morning and evening. In study 2, 24 healthy, young men received 2 identical (850 kcal each) meals in the morning (8:45 h) and evening (18:00 h), of 2 experimental conditions: (i) regular carbohydrate (CH) meals (regular-CH), and (ii) high carbohydrate (high-CH) meals, containing 50 and 80% of energy from CHs, respectively. Serial blood samples were obtained, and the postprandial feelings of hunger, satiety, wanting and liking were assessed. Study 1 revealed a higher hedonic drive to eat in the evening compared to the morning. Study 2 confirmed this diurnal pattern of hedonic appetite regulation and, moreover, showed increased glucose and insulin responses to the evening meal. Postprandial ghrelin and leptin as well as feelings of hunger and satiety were not different between the mealtimes nor between the macronutrient conditions. In line with this, the homeostatic drive to eat was neither affected by the mealtime nor macronutrient composition. Increased the hedonic drive to eat in the evening may represent a vulnerability to palatable food and, thus, energy overconsumption. Together with lower evening glucose tolerance, these findings reflect an adverse metabolic constellation at the end of the day, especially after the ingestion of CH-rich foods.

## 1. Introduction

Overweight and obesity persist as concerning health challenges worldwide, and almost unlimited access to palatable and high-energy food triggers energy overconsumption in most Western societies [1,2]. A high energy intake as well as timing of food intake have been proposed as the main factors modulating metabolic and body weight control in humans [3,4,5]. Although dietary recommendations for weight loss or weight maintenance vary widely in their macronutrient composition, e.g., low-carb or low-fat, they often do not account for the timing of food intake [6,7].

Findings from animal and human studies have shown that a higher proportion of food intake in the evening worsens metabolic control and is associated with a higher body weight [8,9]. Mice fed a high-fat diet during the resting (light) phase gained more weight than the mice fed an isoenergetic diet only during the active (dark) phase [10]. Although the studies in humans are less controlled for the total energy intake, delayed mealtimes are associated with increased weight gain and metabolic derangement [11,12,13]. Cross-sectional data analysis revealed that the main meal intake around midday is associated with a lower risk of being overweight or obese, whereas the main meal intake in the evening hours is associated with a higher risk [11]. Likewise, early eating patterns are associated with improved weight loss and an improvement in metabolic parameters in adults with obesity [14,15] These findings are supported by data showing an essential role for meal timing in modulating the human circadian system as an essential regulator of metabolic homeostasis [16,17]. Another factor, such as chronotype, i.e., the individual circadian phenotype in behavioral and biological rhythms, is influenced by genetic and environmental factors and has also been associated with obesity and chronic diseases [18,19]. It has been recently demonstrated that early meal timing and early chronotype are associated with a reduced appetite and desire for high-fat foods [20]. More recently, the eating speed of a meal has also been associated with the risk of cardiometabolic risk factors in the context of obesity [21]. 

In addition to the time-of-day of food intake, the macronutrient composition impacts the metabolic response to meals across the day. Adults with obesity on a hypoenergetic diet over 4 months achieved better weight-loss maintenance when consuming a breakfast rich in protein and carbohydrates (CHs) than a low-protein–CH breakfast. Consecutively, lower plasma ghrelin and increased satiety feelings were found [22]. Further, a high-fat meal at breakfast and high-CH meal at dinner impaired the postprandial glucose metabolism in non-obese glucose-intolerant men, compared to an inversed order of macronutrient distribution (i.e., high-CH in the morning and high-fat in the evening) [23]. However, there is less knowledge about the potential interaction of meal timing and macronutrient composition on metabolic control and reward-related food intake regulation.

In the present paper, we evaluate the differential and combined effects of meal timing and macronutrient composition on metabolic responses and the homeostatic vs. hedonic drive to eat. First, we conducted an online study (study 1) in healthy young adults to explore the potential time-of-day-dependent differences in homeostatic and hedonic appetite control. After that, we tested the differential effects of meal timing and macronutrient composition on the metabolic responses and homeostatic vs. hedonic appetite regulation in a controlled experimental study (study 2). We hypothesized the time-dependent effects of meal timing on hedonic appetite control with an enhanced drive to eat in the evening as compared to morning, which is more pronounced under a high-CH intake.

## 2. Materials and Methods

Both studies were approved by the Ethics Committee of the University of Luebeck (AZ12-041, 28 April 2017), according to the principles of the Declaration of Helsinki. All the participants gave written informed consent before participation.

### 2.1. Study 1

The homeostatic and hedonic drive to eat were assessed by the liking and wanting paradigm [24] in the morning and the evening, respectively, through an online survey using the web platform SoSci Survey (2006–2015 SoSci Survey GmbH, Munich, Germany). An invitation to participate in this online-based study was sent to all the students at the University of Luebeck, Luebeck, Germany.

A total of 84 young (≥18 years old), normal-weight healthy participants answered the invitation to participate and, thus, were enrolled in the study. After an introductory part informing them about the aim of the study and asking for the general subject characteristics, the participants rated a series of food stimuli using a test—modified for online use—of explicit liking and wanting for foods, as previously reported [24]. In brief, 42 food images differing in energy content (i.e., high- or low-energy) and taste (i.e., non-sweet or sweet) were shown. The food items were classified according to their energy content and separated into non-sweets (i.e., high-energy, fat-rich foods) and sweets (i.e., high-energy, sugar-rich foods). Examples of high-energy non-sweet (HENS) foods were pasta, pizza, burgers, lasagne, French fries, and meat sandwiches. Examples of high-energy sweet (HES) foods were cookies, cakes, pies, pancakes, brownies, and waffles. The participants were asked, ‘How pleasant would you find the taste of this food?’ to assess the liking aspect, and ‘How much do you want some of this food right now?’ for the wanting aspect, respectively. All items were rated on a Likert scale from 1 (‘not at all’) to 5 (‘very much’). The survey was accessible only between 8.00–11.00 h and 18.00–21.00 h for the morning and evening assessments, respectively. Participants had to complete both surveys on the same day. Separate liking and wanting scores were obtained according to three analyzed food categories, i.e., HENS, HES, and low-energy (LE) foods.

### 2.2. Study 2

In this controlled experimental study, healthy normal-weight young men were recruited through public advertisements at the University of Luebeck, and relevant social media channels. The inclusion criteria were healthy adults (≥18 years old), an European background, a body-mass index (BMI) between 18.5–24.9 kg/m^2^, no regular medication, and no specific dietary treatment 3 months before recruitment. The exclusion criteria were current or chronic medical or neurological disorders, high blood pressure, alterations in plasma glucose or lipid metabolism, sleep or mood disorders, shift work, alcohol (>50 g per day) or caffeine (>300 mg per day) consumption, nicotine, and any drug of abuse, and the engagement in competitive sports (e.g., competitive runners). A comprehensive medical history, physical examination, and routine laboratory tests were performed in the eligible subjects during a screening visit.

### 2.3. Experimental Procedures 

**Figure 1:**
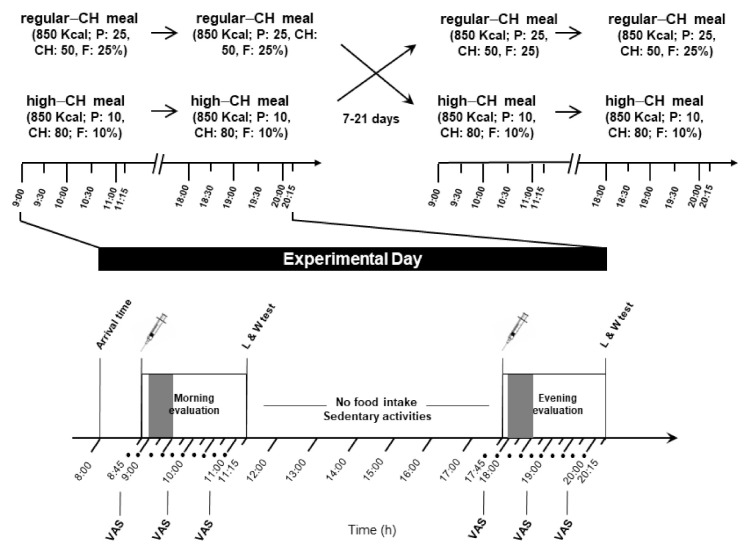
Experimental protocol (study 2). Morning and evening evaluation included the intake of identical meals (850 kcal), either regular or high in carbohydrates (50 and 80% of energy coming from carbohydrates, respectively). Each condition (with high or regular CH meals) was separated by 7 to 21 days. The food intake period is depicted in the gray color bar at both times of the day. In between the sessions, no food intake was allowed except water, and subjects could engage in sedentary activities, such as reading, or watching TV or movies. Approximately two hours after the meal initiation, a computer-based liking and wanting test assessing the homeostatic and hedonic drive to eat was applied; • denotes blood sampling; CH: carbohydrates; P: protein; F: fat; VAS: visual analog scale for subjective feelings of hunger and satiety; and L/W test: liking and wanting test.

A total of 24 young (mean ±standard error of the mean (SEM) age: 24.7 ± 2.0 years, age range: 19–37 years), healthy men with a normal weight (mean BMI 22.6 ± 1.4 kg/m^2^), were enrolled in the study. They were instructed to maintain a regular dietary pattern and sleep routine during the whole study period. The experiments were performed in a randomized, balanced, within-subject design on two separate days spaced at least one week apart (Figure 1).

The procedures were identical during both experimental days, except for the type of served meals. After a 10 h overnight fast with regular 7–8 h of sleep (checked by a structured interview at the beginning of each experimental day), the subjects arrived at 08.00 a.m. at the Institute for Endocrinology and Diabetes of the University of Luebeck. A structured interview assessed the subjective well-being and overall health. For serial blood drawing, a venous cannula was inserted into the non-dominant arm or cubital fossa. At 08.30 a.m., a first blood sample was obtained for fasting blood glucose and hormonal parameters. At 09.00 a.m., the participants received the morning meal, and serial blood samples were taken during the next 120 min until the same protocol was applied at 18.00 p.m. for the evening meal (Figure 1). The participants were supervised by the experimenters during the whole experiment and were allowed to drink water only (<1.5 l total) between the test meals.

### 2.4. Meals

Isoenergetic meals (850 kcal each) were identical within 1 experimental condition but differed in their macronutrient composition between conditions (for served food items, please see Table 1). The macronutrient composition (as a percentage from energy) was 50% CH, 25% protein, and 25% fat for the regular-CH condition, whereas the high-CH condition contained 80% CH, 10% protein, and 10% fat. The energy content and macronutrient composition of the served food items were calculated from manufacturer data and standard software for nutritional analyses (DGE-PC professional 3.3; Stuttgart, Germany). All the meals were prepared and weighed by the experimenters and offered in the same comfortable lab room. The subjects were instructed to eat the whole meal, and the experimenters verified that no food was left after a period of 30 min.

### 2.5. Feelings of Hunger and Appetite

Before the meal initiation and every 60 min thereafter, each subject rated their subjective feelings of hunger, satiety, and overall desire to eat using a 100 mm visual analog scale (VAS) anchored from ‘not at all’ to ‘extremely’. 

### 2.6. Hedonic and Homeostatic Drive to Eat

Two hours after the respective meal, the homeostatic and hedonic drive to eat was assessed by the liking and wanting paradigm for food (as described above in Study 1), which was presented using a personal computer (Matlab, v7.5.0, The MathWorks, Inc., Natick, MA, USA). 

### 2.7. Blood Parameters

Plasma glucose and lactate were measured during the experiments from fluoride plasma (EKF-Diagnostic GmbH, Barleben, Germany). All other blood samples were centrifuged at 4 °C, and the supernatants were stored at −80 °C until analyses. Plasma insulin and cortisol were measured by enzyme-linked immune assays (Immulite, Siemens Healthcare Diagnostics GmbH, Eschborn, Germany) with the following intra (CVintra) and inter (CVinter) coefficients of variation: <5.5% and <7.3% for insulin, and <8.8% and <10.0% for cortisol. Leptin, as well as acylated (active) and non-acylated (total) ghrelin (RIA, Merk Millipore, Darmstadt, Germany), were measured by radioimmunoassays. Data for CVintra and CVinter were <6.2% and <8.3%, <9.5% and 16.2%, and <10.0% and <17.8% for leptin, acylated and non-acylated ghrelin, respectively.

### 2.8. Statistical Analyses

Data are presented as the means ± SEM, unless otherwise indicated, and were analyzed using STATA v.13.1 (StataCorp LLC, College Station, TX, U.S.A.). Figures were processed with GraphPad Prism v. 6 (GraphPad Software, LaJolla, CA, U.S.A.). A one-way analysis of variance (ANOVA) for repeated measures with the ‘time-of-day’ factor was used to compare the morning vs. evening homeostatic and hedonic drive to eat, respectively. The subcategories of the food items were analyzed by Student’s *t*-test, if appropriate. For the glucose and hormonal data (study 2), analyses were based on a three-way repeated-measures ANOVA, with the independent factors of ‘time-of-day’ (morning vs. evening), ‘condition’ (regular- vs. high-CH), and the within-subject factor of ‘time course’ (for the repeated measurements during the day). The Greenhouse–Geisser procedure for the correction of degrees of freedom was used, if appropriate. The insulin resistance was calculated from fasting plasma glucose and insulin using the HOMA model (HOMA-IR: (fasting insulin (mU/L) * fasting glucose (mmol/L)/22.5) [25]. The acylated ghrelin-to-leptin ratio (GLR) was calculated from the plasma active ghrelin and leptin [26]. In addition to the ‘time-of-day’, condition, age, and BMI as covariates, a linear regression model was used to evaluate the relationship between the hedonic and homeostatic drive to eat with HOMA-IR and GLR. The level of significance was set at 5%. 

## 3. Results

### 3.1. Study 1

**Figure 2:**
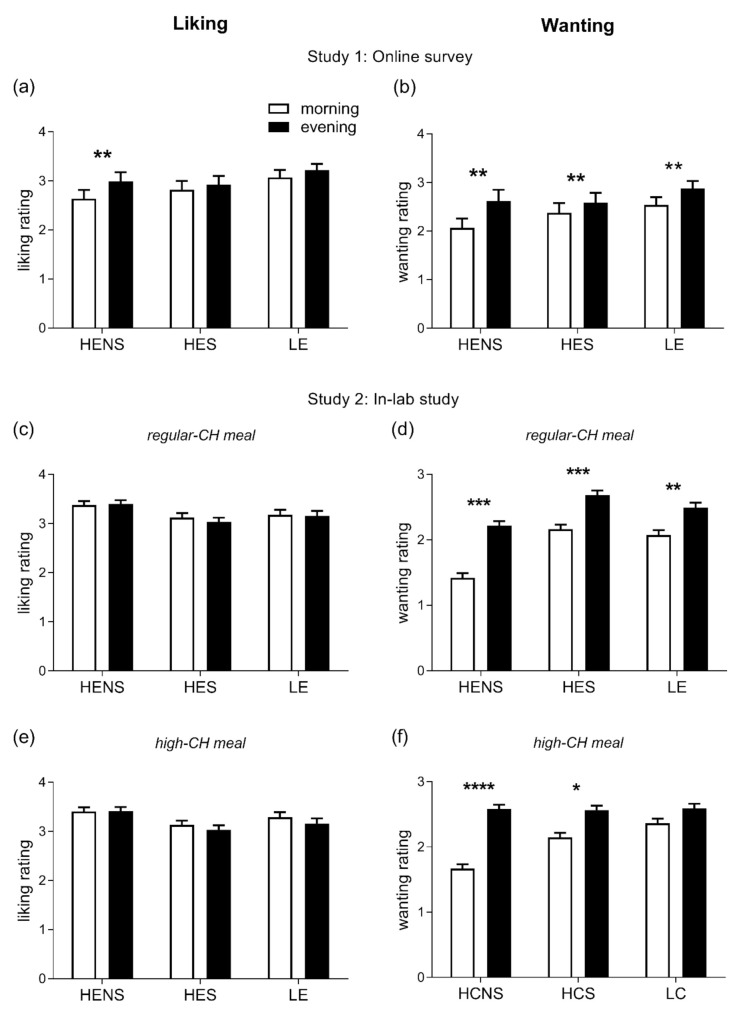
Ratings of liking (**left** column) and wanting (**right** column) for foods. (**a**,**b**): Liking and wanting were evaluated in the morning (8:00–11:00) and evening (18:00–21:00) hours in healthy adults under free-living conditions; differences between morning vs. evening: ** *p* < 0.01. (**c**–**f**): Liking and wanting were evaluated 2 h after the intake of regular-CH (**c**,**d**) and high-CH (**e**,**f**) meals in the morning and evening in healthy adults under laboratory conditions; differences between morning vs. evening: * *p* < 0.05, ** *p* < 0.01, *** *p* < 0.001, **** *p* < 0.0001. Data presented as mean ± SEM. HENS: high-energy non-sweet foods; HES: high-energy sweet foods; LE: low-energy foods; and CH: carbohydrates.

A total of 75 subjects (89.3%) completed the online questionnaire and, thus, the respective data sets were included in the analyses. The participants’ mean age was 24.8 ± 2.0 years (age range: 18.4–38.9 years), mean BMI 21.8 ± 1.5 kg/m^2^, and 42 (56%) were female. The homeostatic drive to eat in general was found to be higher in the evening than in the morning hours (*p* = 0.001, Figure 2a), driven by higher ratings for HENS foods only (*p* = 0.014, for pairwise comparison morning vs. evening). The hedonic drive to eat was significantly enhanced in the evening (*p* = 0.0001, Figure 2b) with increased ratings for all categories of foods, i.e., HENS, HES, and LE (all *p* < 0.05, for pairwise comparisons morning vs. evening).

### 3.2. Study 2

#### 3.2.1. Subjective Feelings of Hunger and Satiety 

**Figure 3:**
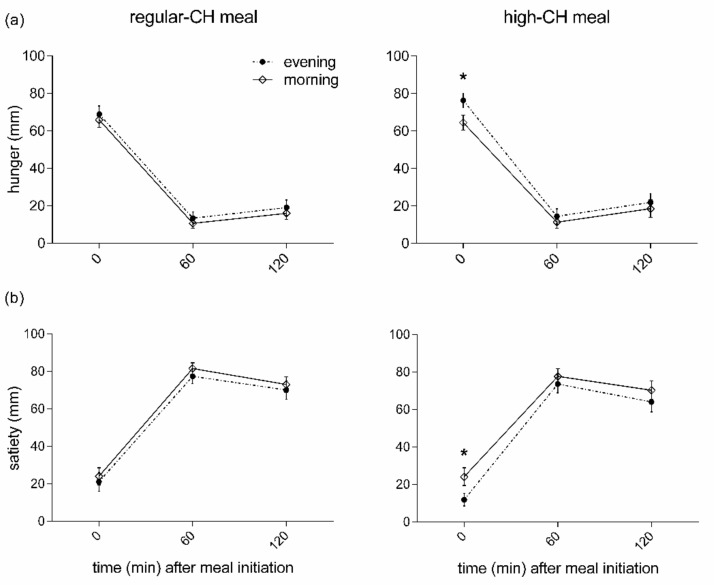
Feelings of hunger (**a**) and satiety (**b**) before and after the intake of regular (**left** columns)- or high (**right** columns)-carbohydrate morning and evening meals. Data presented as mean ± SEM. Differences between both times of the day (morning vs. evening): * *p* < 0.05. CH: carbohydrates.

The subjective pre-prandial feelings of hunger were higher in the evening than the morning, irrespective of macronutrient condition (*p* = 0.007 for ANOVA main effect ‘time-of-day’, Figure 3). In line with these results, an opposite temporal pattern was found for the pre-prandial feelings of satiety with lower values in the evening (*p* = 0.021 for ANOVA main effect ‘time-of-day’, Figure 3). The high-CH meal was less satiating than the regular-CH meal (*p* = 0.023 for ANOVA main effect ‘condition’). Of note, the overall ‘time-of-day’ effect was driven by the pre-prandial ratings (*p* < 0.041 for pairwise comparison). In contrast, all the postprandial ratings, i.e., at the assessment of the homeostatic and hedonic drive to eat for satiety and hunger, were not different between the morning and evening (all *p* > 0.391 for pairwise comparisons).

#### 3.2.2. Homeostatic and Hedonic Drive to Eat 

The homeostatic drive to eat did not differ between the morning and evening and across the meal conditions (ANOVA ‘time-of-day’ and ‘time-of-day x condition’, both *p* > 0.399). However, the hedonic drive to eat was significantly enhanced in the evening (ANOVA ‘time-of-day’ *p* = 0.0001). As seen in study 1, this pattern was confirmed when categorizing for HENS, HES, and LE food items, respectively (all *p* < 0.05, for pairwise comparisons morning vs. evening, Figure 2c–f). When assessing the relation between GLR and HOMA-IR with the homeostatic and hedonic drive to eat, GLR was associated with a homeostatic drive for LE foods (β: 0.15, *p* = 0.001). HOMA-IR was inversely related to a homeostatic drive for LE (β: −0.14, *p* = 0.0005) and positively correlated with high-energy (both HES and HENS) foods (β: 0.26, *p* = 0.0001), respectively. In addition, GLR was positively correlated to the hedonic drive for LE (β: 0.28, *p* = 0.001) and high-energy foods (β: 0.19, *p* = 0.0001). HOMA-IR was positively correlated with a hedonic drive for high-energy foods (β: 0.17, *p* = 0.0001). Of note, the diurnal variation in the hedonic drive to eat did not correlate with circadian fluctuations in the circulating cortisol concentrations (*p* = 0.107).

#### 3.2.3. Glucose Homeostasis

**Figure 4:**
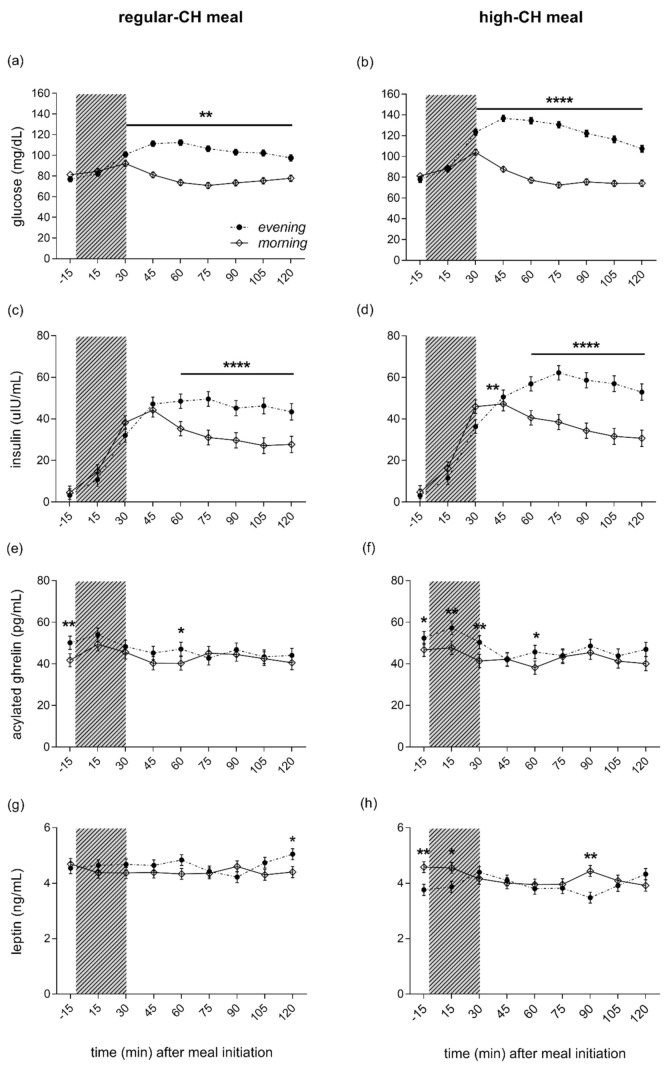
Plasma glucose (**a**,**b**), insulin (**c**,**d**), ghrelin (**e**,**f**), and leptin (**g**,**h**) concentrations before and after the intake of a regular (**left** columns)- and high (**right** columns)-carbohydrate morning and evening meal. The food intake period is depicted as a gray colored bar. Morning (white circles, solid line) and evening (black circles, dashed line) meals. The black line below asterisk denotes all the time points with significant morning vs. evening differences. Data presented as mean ± SEM. Differences between both times of the day (morning vs. evening): * *p* < 0.05, ** *p* < 0.01, **** *p* < 0.0001. CH: carbohydrates.

Fasting plasma glucose, insulin, and peripheral insulin resistance as assessed by HOMA-IR showed lower values in the evening as compared to the morning (ANOVA ‘time-of-day’, all *p* < 0.0001), but were not different across meals (ANOVA ‘time-of-day x condition’, all *p* = 0.415). The evening meal, compared to the morning meal, was consistently followed by more enhanced glucose excursions, starting at 30 min after the meal initiation (ANOVA ‘time-of-day’, *p* = 0.0001, Figure 4a,b). The postprandial glucose response to the meals was more pronounced after the high-CH meal than the regular-CH meal, with a higher and delayed glucose peak after the high-CH meal in the evening (ANOVA ‘time-of-day × condition’, *p* < 0.0001). Plasma insulin mirrored the glucose response with a higher response in the evening (ANOVA ‘time-of-day’, *p* = 0.0001), but with a similar pattern between both meals (ANOVA ‘time-of-day × condition’, *p* = 0.110, Figure 4c,d). Postprandial AUC (0–120 min) for glucose confirmed these findings (ANOVA ‘time-of-day × condition’, *p* < 0.0001, Figure S1). Accordingly, for insulin, a higher AUC was observed in the evening (ANOVA ‘time-of-day’, *p* = 0.0001) with a similar increase across the meals (ANOVA ‘time-of-day × condition’, *p* = 0.236, Figure S1).

#### 3.2.4. Leptin, Ghrelin, and Cortisol

Active ghrelin was higher in the evening than in the morning (ANOVA ‘time-of-day’, *p* = 0.0001), irrespective of the meal condition (ANOVA ‘condition’, *p* = 0.670, Figure 4e,f). In line with the subjective feelings of hunger, this overall ‘time-of-day’ effect was driven by elevated pre-prandial concentrations. At the same time, the postprandial ghrelin dropped to reach comparable levels at 120´ in the morning and evening across both meal conditions (ANOVA ‘time-of-day x condition’, *p* = 0.540). Plasma leptin did not differ between morning and evening hours (ANOVA ‘time-of-day’, *p* = 0.909). However, reduced leptin concentrations were evident after the high-CH meal (ANOVA ‘condition’, *p* = 0.0001), particularly right before and 90 min after the high-CH meal in the evening (ANOVA ‘time-of-day × circadian × meal’, *p* = 0.037, Figure 4g,h). Plasma cortisol showed the expected diurnal variation with higher concentrations in the morning than the evening (ANOVA ‘time-of-day’, *p* = 0.0001, Figure S2). Of note, the time course of cortisol after food intake did not differ between morning and evening, nor between the meal conditions (ANOVA ‘time-of-day × condition × time course’ *p* = 0.375). 

## 4. Discussion

Our data suggest that the time-of-day impacts the regulation of a hedonic appetite in healthy humans. Under both free-living and well-controlled laboratory conditions, the postprandial homeostatic drive for energy-dense foods was not different in the morning and evening. In contrast, the evening hedonic drive was clearly enhanced, despite the comparable feelings of hunger and satiety. Further, the postprandial glucose and insulin responses to isoenergetic meals were exacerbated in the evening, an effect that was further enhanced by a high-CH meal content. 

The diurnal variations in human blood glucose and plasma insulin are well-known [27], with increased and approximately two-fold larger glucose responses after meal ingestion in the evening than in the morning [28]. Insulin sensitivity and glucose tolerance show a diurnal rhythm [29], with maximal insulin sensitivity in the early morning, followed by a decrease throughout the day [28,30]. Our data confirm a state of reduced insulin sensitivity in the evening, with higher and more prolonged glucose and insulin excursions after an evening meal compared to an isoenergetic morning meal. This effect was even more pronounced when the carbohydrate proportion of the test meals was increased. Focusing on dinner time, it has been shown that a late dinner at 22:00 p.m. impairs glucose metabolism and fat oxidation, compared to a regular dinner at 18:00 p.m. in healthy young adults [31]. Further data indicate increased glucose excursions after meals in the evening time, compared to the morning time, even when testing the identical low-glycemic content meals are offered [32,33]. Others have added support to the time-of-day effect of meal timing on glucose metabolism and blood metabolites, confirming an altered evening glucose metabolism together with enhanced incretin and overall blood metabolites responses in the morning hours [34]. This diurnal variation of glucose homeostasis might explain the epidemiological studies that early meal-timing is inversely associated with total energy intake and risk for obesity and diabetes [35,36].

Assessing the liking and wanting for food stimuli under the postprandial conditions at both times of the day, the hedonic, but not the homeostatic, drive for energy-rich foods was enhanced in the evening compared to the morning hours. This pattern was robust under both free-living and well-controlled laboratory settings. Liking and wanting for foods are two separate processes modulating food reward [37]. Whereas liking reflects the sensory pleasure experience (palatability) and represents the homeostatic drive to eat, wanting reflects the motivation related to appetite (incentive) that translates into action, i.e., the actual desire for food-reward [38]. The functional imaging data shows that cerebral structures, such as the nucleus accumbens and ventral pallidum, are differentially and critically involved in homeostatic and hedonic appetite control, suggesting a neural dissociation of liking and wanting for foods and food-related cues [39].

The effect of both the type of food (high- vs. low-energy foods) and time-of-day (morning vs. evening) on brain activation in lean young women [40], was previously evaluated by functional magnetic resonance imaging indicating the reduced activation of several brain areas related to visual stimuli, food-stimuli processing, and reward, respectively, in the evening hours. At a first glance, these data contrast with our results of similar a homeostatic but increased hedonic drive for foods following a controlled feeding state and under similar postprandial feelings of hunger and satiety. However, the reduced activation of reward-related brain areas could lead to increased motivation for foods later during the day and eventually exacerbate food intake in the evening, as discussed by the authors [40]. Masterson and co-workers also found increased thoughts about foods and an enhanced desire to eat in the evening [40]. These data are supported by others showing peaked appetite/hunger feelings in the late part of the day [41] and a reduced satiating effect of a meal across the day [42]. The observational data indicate a time-of-day effect on the desire to eat with increased levels for sweets and salty snacks later in the day [43]. Wanting for foods can arise even in the absence of an increased liking, probably mediated by elevated dopamine levels in the mesolimbic areas [44], and excessive wanting is observed in some cases of obesity [45]. Thus, our results support the evidence that the time-of-day represents a key factor influencing hedonic appetite regulation. In this context, existing data confirm increased hunger and lower feelings of satiety in the afternoon, concomitant with higher ghrelin and lower peptide YY levels in patients with obesity or binge-eating disorder [46].

Recent evidence supports leptin’s and ghrelin’s role in acting as critical factors regulating the hedonic control of food intake. Ghrelin increases the incentive value for food reward [47], and higher fasting ghrelin was associated with stronger appetite feelings [48]. The subcutaneous administration of ghrelin acutely stimulates the hedonic responses and activation of corticolimbic reward-cognitive systems during food evaluations, with increased hippocampus activation in response to food images [47]. Leptin involves food reward processes as the activation of the leptin receptor inhibits the activity of neurons in the ventral tegmental area (VTA) [49]. Leptin can decrease feeding-induced dopamine release in the ventral striatum [50]. Here, we report increased fasting ghrelin, lower leptin, and higher GLR in the evening, which are in concordance with exacerbated feelings of hunger and lower satiety before the evening meal, particularly after a high-CH morning meal. It is worth noting that the postprandial (120 min) ghrelin and leptin concentrations, as well as the subjective feelings of hunger and satiety, were similar after food intake in morning and evening hours. 

We further explored the potential effect of GLR and HOMA-IR on the hedonic drive to eat. Higher HOMA-IR was related to an increased hedonic drive for foods, whereas higher GLR associates to both an increased homeostatic and hedonic drive to eat. Our findings highlight the interaction between peripheral homeostatic signals, such as insulin and leptin, and the hedonic aspects of appetite control. Farooqi et al. [51] reported an increased activation in the ventral striatum in response to food images in leptin-deficient humans. In contrast, leptin-replacement decreased not only the feelings of hunger, but also the activation of mesolimbic areas and liking ratings in response to the food images. Regarding the role of insulin in appetite regulation, there is emerging evidence that insulin modulates the brain areas related to food reward [52]. Additionally, the subjects with insulin resistance show altered insulin brain regulation with reduced valuation to food cues in the VTA and nucleus accumbens [53]. These findings highlight the impact of increasing insulin resistance, as seen in the prediabetes or diabetes, on reward-driven appetite regulation [54]. 

Our study has limitations that we would like to mention. We evaluated a group of young, normal-weight men. Thus, our findings cannot be extrapolated to other age, sex, and BMI groups. Since we evaluated healthy subjects with normal glucose metabolism, our results will require further confirmation in subjects with altered metabolic regulation, such as patients with obesity, insulin resistance, or type 2 diabetes. However, we believe that the experimental design and the well-controlled protocol and meals contribute to a better understanding of the time-of-day effect on human energy homeostasis. The combination of real-life and experimental settings further supports our findings. 

## 5. Conclusions

To summarize, our findings show an enhanced hedonic drive to eat after an evening meal despite stable homeostatic appetite control, which may increase the vulnerability for energy overconsumption at the end of the day. Together with lower insulin sensitivity, these findings reflect an adverse metabolic constellation in the evening, especially after the ingestion of CH-rich foods. Further studies should explore the impact of the circadian clock on humans’ hedonic drive to eat and its relevance for pathological conditions, such as obesity and obesity-related metabolic disorders.

## Figures and Tables

**Table 1:** Macronutrient composition of regular-CH and high-CH meals was as follows: regular-CH: 25, 50, and 25% of energy content coming from protein, CH, and fat, respectively; high-CH meal: 10, 80, and 10% of energy content coming from protein, CH, and fat, respectively. Both meals had an identical energy content (850 kcal), resulting in a total daily energy intake of 1700 kcal. * Bread with 26% whole wheat. CH: carbohydrates.

Regular-CH Meal	g/mL	High-CH Meal	g/mL
Wheat bread	70 g	Whole wheat bread *	88 g
Whole wheat bread *	70 g	Skim milk, 1.5% fat	130 mL
Skim milk, 1.5% fat	240 mL	Smoked ham	20 g
Skim yogurt, 1.5% fat	250 mL	Cream cheese	5 g
Smoked ham	40 g	Apple juice	200 mL
Cream cheese	30 g	Banana	225 g
Camembert cheese	40 g	Apple	225 g
Banana	120 g	Strawberry jam	30 g
Water	200 mL	Water	110 mL

## Data Availability

The data presented in this study are available upon request from the corresponding author.

## Supplementary Materials

The following supporting information can be downloaded at: https://www.mdpi.com/article/10.3390/nu14030562/s1, Figure S1: glucose (a) and insulin (b) area under the curve after the intake of a regular- and high-carbohydrate morning and evening meal; Figure S2: plasma cortisol concentration before and after the intake of a regular (left columns)- and high (right columns)-carbohydrate morning and evening meal.

## References

[1] (2017). NCD Risk Factor Collaboration (NCD-RisC) Worldwide Trends in Body-Mass Index, Underweight, Overweight, and Obesity from 1975 to 2016: A Pooled Analysis of 2416 Population-Based Measurement Studies in 128·9 Million Children, Adolescents, and Adults. Lancet Lond. Engl..

[2] Kelly T., Yang W., Chen C.S., Reynolds K., He J. (2008). Global Burden of Obesity in 2005 and Projections to 2030. Int. J. Obes..

[3] Garaulet M., Gómez-Abellán P. (2014). Timing of Food Intake and Obesity: A Novel Association. Physiol. Behav..

[4] Mattson M.P., Allison D.B., Fontana L., Harvie M., Longo V.D., Malaisse W.J., Mosley M., Notterpek L., Ravussin E., Scheer F.A.J.L. (2014). Meal Frequency and Timing in Health and Disease. Proc. Natl. Acad. Sci. USA.

[5] St-Onge M.-P., Ard J., Baskin M.L., Chiuve S.E., Johnson H.M., Kris-Etherton P., Varady K. (2017). American Heart Association Obesity Committee of the Council on Lifestyle and Cardiometabolic Health; Council on Cardiovascular Disease in the Young; Council on Clinical Cardiology; and Stroke Council Meal Timing and Frequency: Implications for Cardiovascular Disease Prevention: A Scientific Statement from the American Heart Association. Circulation.

[6] Ge L., Sadeghirad B., Ball G.D.C., da Costa B.R., Hitchcock C.L., Svendrovski A., Kiflen R., Quadri K., Kwon H.Y., Karamouzian M. (2020). Comparison of Dietary Macronutrient Patterns of 14 Popular Named Dietary Programmes for Weight and Cardiovascular Risk Factor Reduction in Adults: Systematic Review and Network Meta-Analysis of Randomised Trials. BMJ.

[7] Hession M., Rolland C., Kulkarni U., Wise A., Broom J. (2009). Systematic Review of Randomized Controlled Trials of Low-Carbohydrate vs. Low-Fat/Low-Calorie Diets in the Management of Obesity and Its Comorbidities. Obes. Rev..

[8] Hibi M., Masumoto A., Naito Y., Kiuchi K., Yoshimoto Y., Matsumoto M., Katashima M., Oka J., Ikemoto S. (2013). Nighttime Snacking Reduces Whole Body Fat Oxidation and Increases LDL Cholesterol in Healthy Young Women. Am. J. Physiol. Regul. Integr. Comp. Physiol..

[9] De Castro J.M. (2004). The Time of Day of Food Intake Influences Overall Intake in Humans. J. Nutr..

[10] Arble D.M., Bass J., Laposky A.D., Vitaterna M.H., Turek F.W. (2009). Circadian Timing of Food Intake Contributes to Weight Gain. Obes. Silver Spring.

[11] Wang J.B., Patterson R.E., Ang A., Emond J.A., Shetty N., Arab L. (2014). Timing of Energy Intake during the Day Is Associated with the Risk of Obesity in Adults. J. Hum. Nutr. Diet..

[12] McHill A.W., Phillips A.J., Czeisler C.A., Keating L., Yee K., Barger L.K., Garaulet M., Scheer F.A., Klerman E.B. (2017). Later Circadian Timing of Food Intake Is Associated with Increased Body Fat. Am. J. Clin. Nutr..

[13] Bandín C., Scheer F.A.J.L., Luque A.J., Avila-Gandía V., Zamora S., Madrid J.A., Gómez-Abellán P., Garaulet M. (2015). Meal Timing Affects Glucose Tolerance, Substrate Oxidation and Circadian-Related Variables: A Randomized, Crossover Trial. Int. J. Obes..

[14] Garaulet M., Gómez-Abellán P., Alburquerque-Béjar J.J., Lee Y.-C., Ordovás J.M., Scheer F.A.J.L. (2013). Timing of Food Intake Predicts Weight Loss Effectiveness. Int. J. Obes..

[15] Jakubowicz D., Barnea M., Wainstein J., Froy O. (2013). High Caloric Intake at Breakfast vs. Dinner Differentially Influences Weight Loss of Overweight and Obese Women. Obes. Silver Spring.

[16] Wehrens S.M.T., Christou S., Isherwood C., Middleton B., Gibbs M.A., Archer S.N., Skene D.J., Johnston J.D. (2017). Meal Timing Regulates the Human Circadian System. Curr. Biol..

[17] Lopez-Minguez J., Gómez-Abellán P., Garaulet M. (2019). Timing of Breakfast, Lunch, and Dinner. Effects on Obesity and Metabolic Risk. Nutrients.

[18] Almoosawi S., Vingeliene S., Gachon F., Voortman T., Palla L., Johnston J.D., Van Dam R.M., Darimont C., Karagounis L.G. (2019). Chronotype: Implications for Epidemiologic Studies on Chrono-Nutrition and Cardiometabolic Health. Adv. Nutr..

[19] Muscogiuri G., Barrea L., Aprano S., Framondi L., Di Matteo R., Altieri B., Laudisio D., Pugliese G., Savastano S., Colao A. (2021). Chronotype and Cardio Metabolic Health in Obesity: Does Nutrition Matter?. Int. J. Food Sci. Nutr..

[20] Beaulieu K., Oustric P., Alkahtani S., Alhussain M., Pedersen H., Quist J.S., Færch K., Finlayson G. (2020). Impact of Meal Timing and Chronotype on Food Reward and Appetite Control in Young Adults. Nutrients.

[21] Barrea L., Vetrani C., Verde L., Napolitano B., Savastano S., Colao A., Muscogiuri G. (2021). “Forever Young at the Table”: Metabolic Effects of Eating Speed in Obesity. J. Transl. Med..

[22] Jakubowicz D., Froy O., Wainstein J., Boaz M. (2012). Meal Timing and Composition Influence Ghrelin Levels, Appetite Scores and Weight Loss Maintenance in Overweight and Obese Adults. Steroids.

[23] Kessler K., Hornemann S., Petzke K.J., Kemper M., Kramer A., Pfeiffer A.F.H., Pivovarova O., Rudovich N. (2017). The Effect of Diurnal Distribution of Carbohydrates and Fat on Glycaemic Control in Humans: A Randomized Controlled Trial. Sci. Rep..

[24] Finlayson G., King N., Blundell J.E. (2007). Is It Possible to Dissociate “liking” and “Wanting” for Foods in Humans? A Novel Experimental Procedure. Physiol. Behav..

[25] Matthews D.R., Hosker J.P., Rudenski A.S., Naylor B.A., Treacher D.F., Turner R.C. (1985). Homeostasis Model Assessment: Insulin Resistance and Beta-Cell Function from Fasting Plasma Glucose and Insulin Concentrations in Man. Diabetologia.

[26] Priego T., Sánchez J., Picó C., Palou A. (2009). Sex-Associated Differences in the Leptin and Ghrelin Systems Related with the Induction of Hyperphagia under High-Fat Diet Exposure in Rats. Horm. Behav..

[27] Malherbe C., De Gasparo M., De Hertogh R., Hoet J.J. (1969). Circadian Variations of Blood Sugar and Plasma Insulin Levels in Man. Diabetologia.

[28] Van Cauter E., Polonsky K.S., Scheen A.J. (1997). Roles of Circadian Rhythmicity and Sleep in Human Glucose Regulation. Endocr. Rev..

[29] Jarrett R.J., Keen H. (1970). Further Observations on the Diurnal Variation in Oral Glucose Tolerance. Br. Med. J..

[30] Van Cauter E., Désir D., Decoster C., Féry F., Balasse E.O. (1989). Nocturnal Decrease in Glucose Tolerance during Constant Glucose Infusion. J. Clin. Endocrinol. Metab..

[31] Gu C., Brereton N., Schweitzer A., Cotter M., Duan D., Børsheim E., Wolfe R.R., Pham L.V., Polotsky V.Y., Jun J.C. (2020). Metabolic Effects of Late Dinner in Healthy Volunteers—A Randomized Crossover Clinical Trial. J. Clin. Endocrinol. Metab..

[32] Leung G.K.W., Huggins C.E., Bonham M.P. (2017). Effect of Meal Timing on Postprandial Glucose Responses to a Low Glycemic Index Meal: A Crossover Trial in Healthy Volunteers. Clin. Nutr..

[33] Gibbs M., Harrington D., Starkey S., Williams P., Hampton S. (2014). Diurnal Postprandial Responses to Low and High Glycaemic Index Mixed Meals. Clin. Nutr..

[34] Takahashi M., Ozaki M., Kang M.-I., Sasaki H., Fukazawa M., Iwakami T., Lim P., Kim H.-K., Aoyama S., Shibata S. (2018). Effects of Meal Timing on Postprandial Glucose Metabolism and Blood Metabolites in Healthy Adults. Nutrients.

[35] De Castro J.M. (2007). The Time of Day and the Proportions of Macronutrients Eaten Are Related to Total Daily Food Intake. Br. J. Nutr..

[36] Almoosawi S., Prynne C.J., Hardy R., Stephen A.M. (2013). Time-of-Day and Nutrient Composition of Eating Occasions: Prospective Association with the Metabolic Syndrome in the 1946 British Birth Cohort. Int. J. Obes..

[37] Berridge K.C., Robinson T.E., Aldridge J.W. (2009). Dissecting Components of Reward: ‘liking’, ‘wanting’, and Learning. Curr. Opin. Pharmacol..

[38] Berridge K.C. (1996). Food Reward: Brain Substrates of Wanting and Liking. Neurosci. Biobehav. Rev..

[39] Jiang T., Soussignan R., Schaal B., Royet J.P. (2013). Reward for Food Odors: An FMRI Study of Liking and Wanting as a Function of Metabolic State and BMI. Soc. Cogn. Affect. Neurosci..

[40] Masterson T.D., Kirwan C.B., Davidson L.E., LeCheminant J.D. (2016). Neural Reactivity to Visual Food Stimuli Is Reduced in Some Areas of the Brain during Evening Hours Compared to Morning Hours: An FMRI Study in Women. Brain Imaging Behav..

[41] Scheer F.A.J.L., Morris C.J., Shea S.A. (2013). The Internal Circadian Clock Increases Hunger and Appetite in the Evening Independent of Food Intake and Other Behaviors. Obesity.

[42] De Castro J.M. (1987). Circadian Rhythms of the Spontaneous Meal Pattern, Macronutrient Intake, and Mood of Humans. Physiol. Behav..

[43] Reichenberger J., Richard A., Smyth J.M., Fischer D., Pollatos O., Blechert J. (2018). It’s Craving Time: Time of Day Effects on Momentary Hunger and Food Craving in Daily Life. Nutrition.

[44] Berridge K.C., Ho C.Y., Richard J.M., Difeliceantonio A.G. (2010). The Tempted Brain Eats: Pleasure and Desire Circuits in Obesity and Eating Disorders. Brain Res..

[45] Morales I., Berridge K.C. (2020). “Liking” and “Wanting” in Eating and Food Reward: Brain Mechanisms and Clinical Implications. Physiol. Behav..

[46] Carnell S., Grillot C., Ungredda T., Ellis S., Mehta N., Holst J., Geliebter A. (2018). Morning and Afternoon Appetite and Gut Hormone Responses to Meal and Stress Challenges in Obese Individuals with and without Binge Eating Disorder. Int. J. Obes..

[47] Skibicka K.P., Hansson C., Egecioglu E., Dickson S.L. (2012). Role of Ghrelin in Food Reward: Impact of Ghrelin on Sucrose Self-Administration and Mesolimbic Dopamine and Acetylcholine Receptor Gene Expression. Addict. Biol..

[48] Kroemer N.B., Krebs L., Kobiella A., Grimm O., Pilhatsch M., Bidlingmaier M., Zimmermann U.S., Smolka M.N. (2013). Fasting Levels of Ghrelin Covary with the Brain Response to Food Pictures. Addict. Biol..

[49] Krügel U., Schraft T., Kittner H., Kiess W., Illes P. (2003). Basal and Feeding-Evoked Dopamine Release in the Rat Nucleus Accumbens Is Depressed by Leptin. Eur. J. Pharmacol..

[50] Fulton S., Pissios P., Manchon R.P., Stiles L., Frank L., Pothos E.N., Maratos-Flier E., Flier J.S. (2006). Leptin Regulation of the Mesoaccumbens Dopamine Pathway. Neuron.

[51] Farooqi I.S., Bullmore E., Keogh J., Gillard J., O’Rahilly S., Fletcher P.C. (2007). Leptin Regulates Striatal Regions and Human Eating Behavior. Science.

[52] Davis J.F., Choi D.L., Benoit S.C. (2010). Insulin, Leptin and Reward. Trends Endocrinol. Metab..

[53] Tiedemann L.J., Schmid S.M., Hettel J., Giesen K., Francke P., Büchel C., Brassen S. (2017). Central Insulin Modulates Food Valuation via Mesolimbic Pathways. Nat. Commun..

[54] Lutter M., Nestler E.J. (2009). Homeostatic and Hedonic Signals Interact in the Regulation of Food Intake. J. Nutr..

