# Supplementary material for: Meal Timing and Macronutrient Composition Modulate Human Metabolism and Reward-Related Drive to Eat
Source: Nutrients. 2022 Jan 27;14(3):562. doi: 10.3390/nu14030562 (PMC8839823; doi:10.3390/nu14030562)

Supplementary Materials

Figure S1. Glucose (**a**) and insulin (**b**) area under the curve after the intake of a regular- and high-carbohydrate morning and evening meal. Data presented as mean ± SEM. In (**a**): **** $p < 0.00001$ for ANOVA 'time-of-day × condition'; in (**b**): ****$p < 0.0001$ for ANOVA 'time-of-day'. CH: carbohydrates.

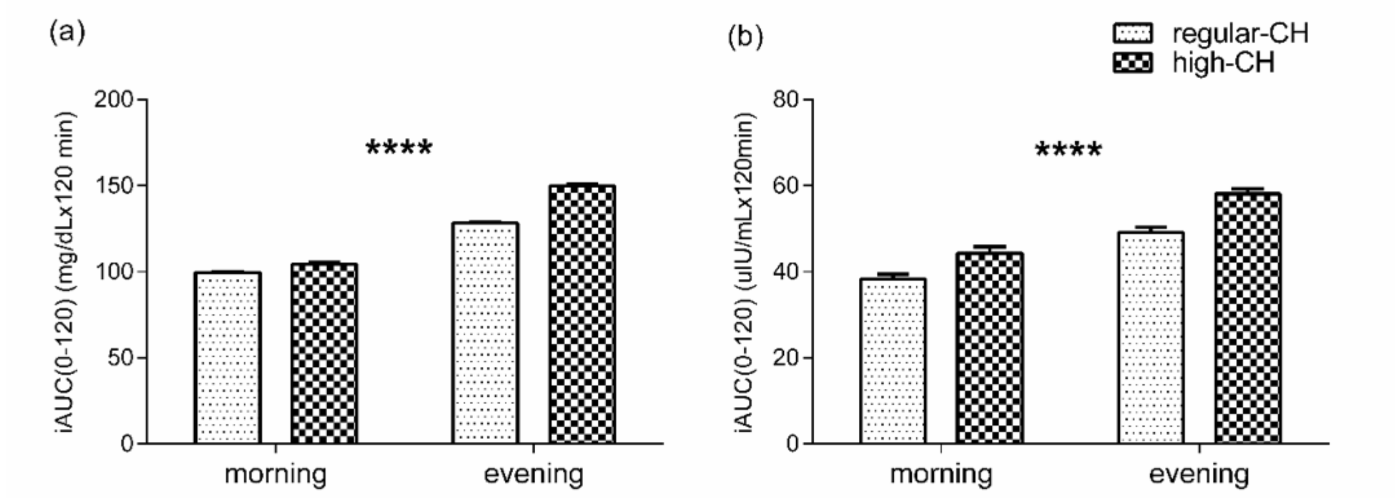

Figure S2. Plasma cortisol concentration before and after the intake of a regular- (left columns, (**a**), and high- (right columns, (**b**), carbohydrate morning and evening meal. Food intake period is depicted as a grey colored bar. Morning (white circles, solid line) and evening (black circles, dashed line) meal. Blackline below asterisk denotes all time points with significant morning vs. evening differences (in (**a**) all morning vs. evening comparison $p < 0.01$). Data presented as mean ± SEM. Differences between both times of the day (morning vs. evening): * $p < 0.05$, ***$p < 0.001$. CH: carbohydrates.

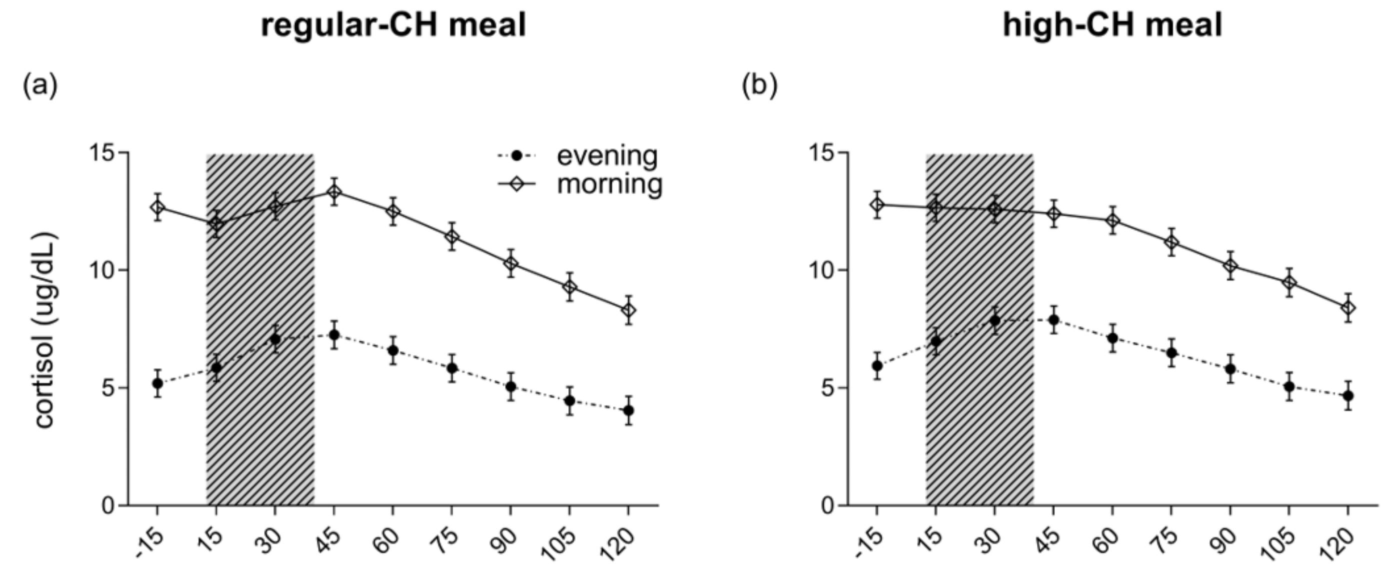

Supplement: Supplementary file 1 [file nutrients-14-00562-s001.zip › Supplementary.pdf]
